# Development and validation of the BRIGHTLIGHT Survey, a patient-reported experience measure for young people with cancer

**DOI:** 10.1186/s12955-015-0312-7

**Published:** 2015-07-28

**Authors:** Rachel M. Taylor, Lorna A. Fern, Anita Solanki, Louise Hooker, Anna Carluccio, Julia Pye, David Jeans, Tom Frere–Smith, Faith Gibson, Julie Barber, Rosalind Raine, Dan Stark, Richard Feltbower, Susie Pearce, Jeremy S. Whelan

**Affiliations:** Cancer Clinical Trials Unit University College London Hospitals NHS Foundation Trust, London, UK; School of Health & Social Care, London South Bank University, London, UK; NIHR University College London Hospitals Biomedical Research Centre, London, UK; University Hospitals of Southampton NHS Foundation Trust, Southampton, UK; Social Research Institute, Ipsos MORI, London, UK; Great Ormond Street Hospital for Children NHS Foundation Trust, London, UK; Department of Statistical Science, University College London, London, UK; Department of Applied Health Research, University College London, London, UK; Leeds Institute of Cancer and Pathology, University of Leeds, Leeds, UK; Division of Epidemiology & Biostatistics, School of Medicine, University of Leeds, Leeds, UK

**Keywords:** Teenager, Young adult, Cancer, Experience, BRIGHTLIGHT

## Abstract

**Background:**

Patient experience is increasingly used as an indicator of high quality care in addition to more traditional clinical end–points. Surveys are generally accepted as appropriate methodology to capture patient experience. No validated patient experience surveys exist specifically for adolescents and young adults (AYA) aged 13–24 years at diagnosis with cancer. This paper describes early work undertaken to develop and validate a descriptive patient experience survey for AYA with cancer that encompasses both their cancer experience and age-related issues. We aimed to develop, with young people, an experience survey meaningful and relevant to AYA to be used in a longitudinal cohort study (BRIGHTLIGHT), ensuring high levels of acceptability to maximise study retention.

**Methods:**

A three-stage approach was employed: Stage 1 involved developing a conceptual framework, conducting literature/Internet searches and establishing content validity of the survey; Stage 2 confirmed the acceptability of methods of administration and consisted of four focus groups involving 11 young people (14–25 years), three parents and two siblings; and Stage 3 established survey comprehension through telephone-administered cognitive interviews with a convenience sample of 23 young people aged 14–24 years.

**Result:**

Stage 1: Two-hundred and thirty eight questions were developed from qualitative reports of young people’s cancer and treatment-related experience. Stage 2: The focus groups identified three core themes: (i) issues directly affecting young people, e.g. impact of treatment-related fatigue on ability to complete survey; (ii) issues relevant to the actual survey, e.g. ability to answer questions anonymously; (iii) administration issues, e.g. confusing format in some supporting documents. Stage 3: Cognitive interviews indicated high levels of comprehension requiring minor survey amendments.

**Conclusion:**

Collaborating with young people with cancer has enabled a survey of to be developed that is both meaningful to young people but also examines patient experience and outcomes associated with specialist cancer care. Engagement of young people throughout the survey development has ensured the content appropriately reflects their experience and is easily understood. The BRIGHTLIGHT survey was developed for a specific research project but has the potential to be used as a TYA cancer survey to assess patient experience and the care they receive.

## Background

BRIGHTLIGHT is a National Institute for Health Research (NIHR) funded project evaluating cancer services for adolescents and young adults (AYA) aged 13–24 years at the time of diagnosis in England (project reference: RP-PG-1209-10013). Specialist cancer services in the UK for this population have developed based on the philosophy that receipt of age-appropriate care during treatment may enable young people to continue with their lives and preserve normal life-stage development, both during and after treatment [1]. To date, there has been no national evaluation of specialist services for young people with cancer across the 13 – 24 year age range. Smaller, single centre studies have shown that young people describe their experience of specialist young person’s care as ‘better’ [2–4]; however, no study has formally identified which outcomes may be affected by care in a specialist young person’s cancer service. Central to BRIGHTLIGHT is the evaluation of cancer and healthcare services from the young person’s perspective with data being collected prospectively by a commercial research company, Ipsos MORI (http://www.ipsos-mori.com/researchspecialisms/socialresearch.aspx), through five surveys (over three years) in a newly formed cohort of young people with cancer.

In 2008, Government policy in the United Kingdom (UK) established the value of ascertaining the patient perspective of the quality of clinical care within the National Health Service (NHS) [5]. This signalled a move from simply assessing quality of care according to clinical or operational metrics, such as survival or length of inpatient stay, to evaluating quality based on other outcomes that also matter to patients and which only they can truly report [6]. Central to this policy are national patient experience surveys and questionnaires for use in acute inpatient care, accident and emergency, maternity care and mental health services. In 2010 this was extended to include a cancer specific experience survey: the National Cancer Patient Experience Survey (NCPES). While these have provided valuable information in guiding healthcare policy and driving improvements in services, the content of these surveys reflect their aim – to assess experience according to the NHS Patient Experience Framework [7]. This information could be used to evaluate cancer services for young people aged 16–24 years (the lower age of inclusion in the NCPES is 16 years), however this would not capture the benefits proposed in the philosophy underpinning specialist AYA cancer care: to meet the needs of young people at a transitional stage of life that not only improves survival but also enables the best quality of life in later adulthood [8, 9]. Our aim was therefore to develop a survey that could capture the impact of care through the life course and reflect the lived experience. Our ideal methodology would have been through an in-depth qualitative study but the resources were not available for the scale of such study required to reflect the variance in this heterogeneous population across England. The challenge was therefore to accurately capture experience through quantitative means.

**Fig. 1:**
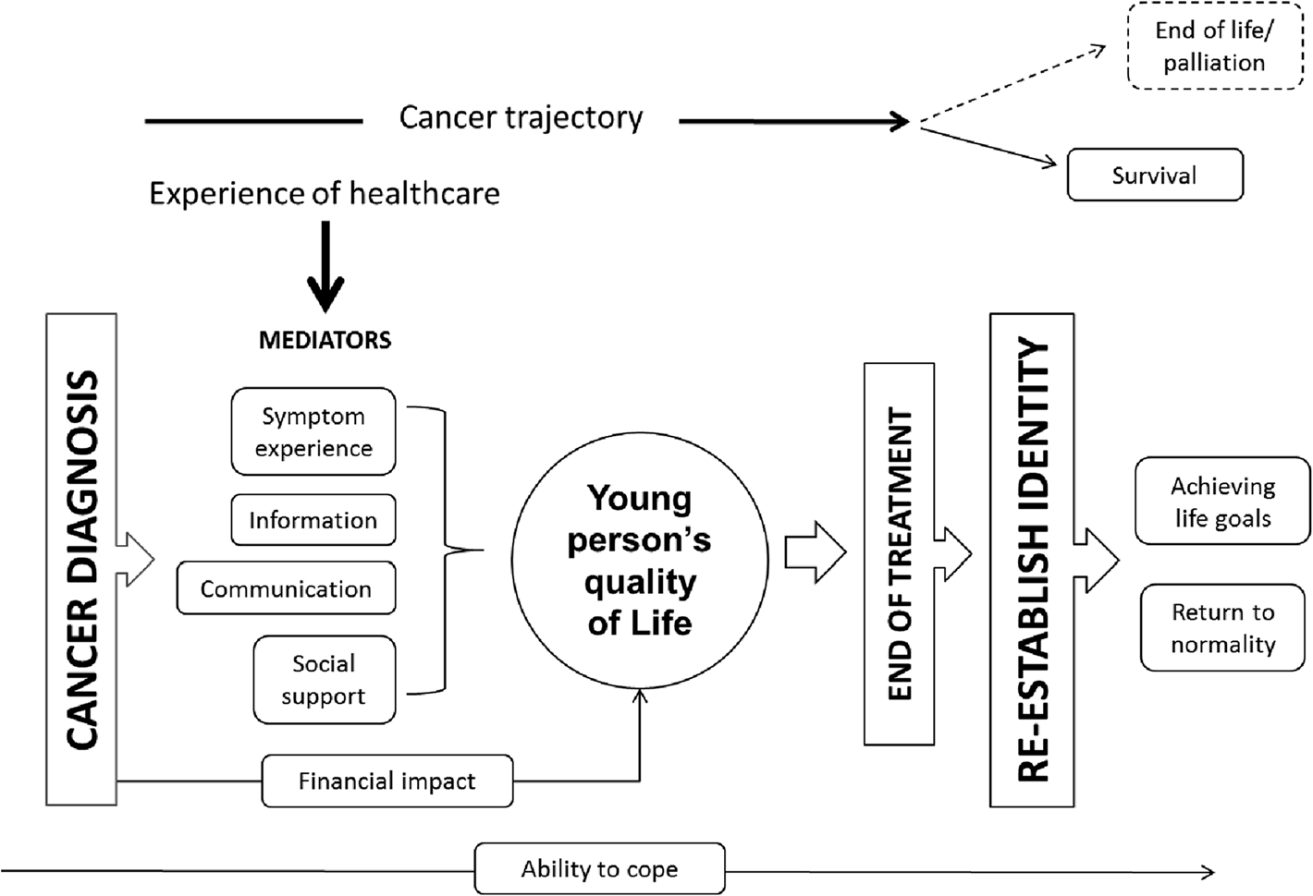
Conceptual framework of teenage and young adult experience of cancer [10]

A key strategy to confirm acceptability of a longitudinal survey to young people with cancer was ensuring it reflected what was important to them. As such, we involved young people in development work for BRIGHTLIGHT which began in 2009 in collaboration with the National Cancer Research Institutes Teenage and Young Adult Clinical Studies Group Core Consumer Group (NCRI TYA CSG CCG). The group comprised of five young people with a previous cancer diagnosis who were trained in research methods [10]. They worked alongside the academic and clinical team advising on study design and were integral in determining the key features of the survey. This feasibility work included: an exploration of young people’s experiences of cancer through a review of the literature [11]; and primary data collection with the NCRI TYA CSG CCG acting as peer interviewers to explore in more detail young people’s experience of cancer [10]. The core consumer group then presented the key themes identified from work back to a wider group of young people for further feedback (https://jtvcancersupport.com/2011/03/fysot-11-ncri-research-for-you/). This work informed the development of a conceptual framework on which to base the BRIGHTLIGHT Survey (Fig. 1).

Involving young people early in the process of survey development highlighted an important perception of young people that they needed to be seen as more than their cancer and the proposed the BRIGHTLIGHT survey would need to reflect more than just cancer-related issues. However, as an evaluation of cancer services it needed to link to the NHS Patient Experience Framework to ensure the results would reflect the priorities of the NHS [7].

In addition issues other than the content of the survey were highlighted by young people as being important and needed to be taken into consideration during survey development. This included format of literature introducing the survey to young people; wanting variation in response formats; the mode of survey administration; and time necessary to complete the survey [12]. Furthermore, while the main focus of BRIGHTLIGHT was patient experience, it was also acknowledged that standard outcome measures should be included to better quantify change over time. Potential outcome measures were also identified during the feasibility work [12].

**Fig. 2:**
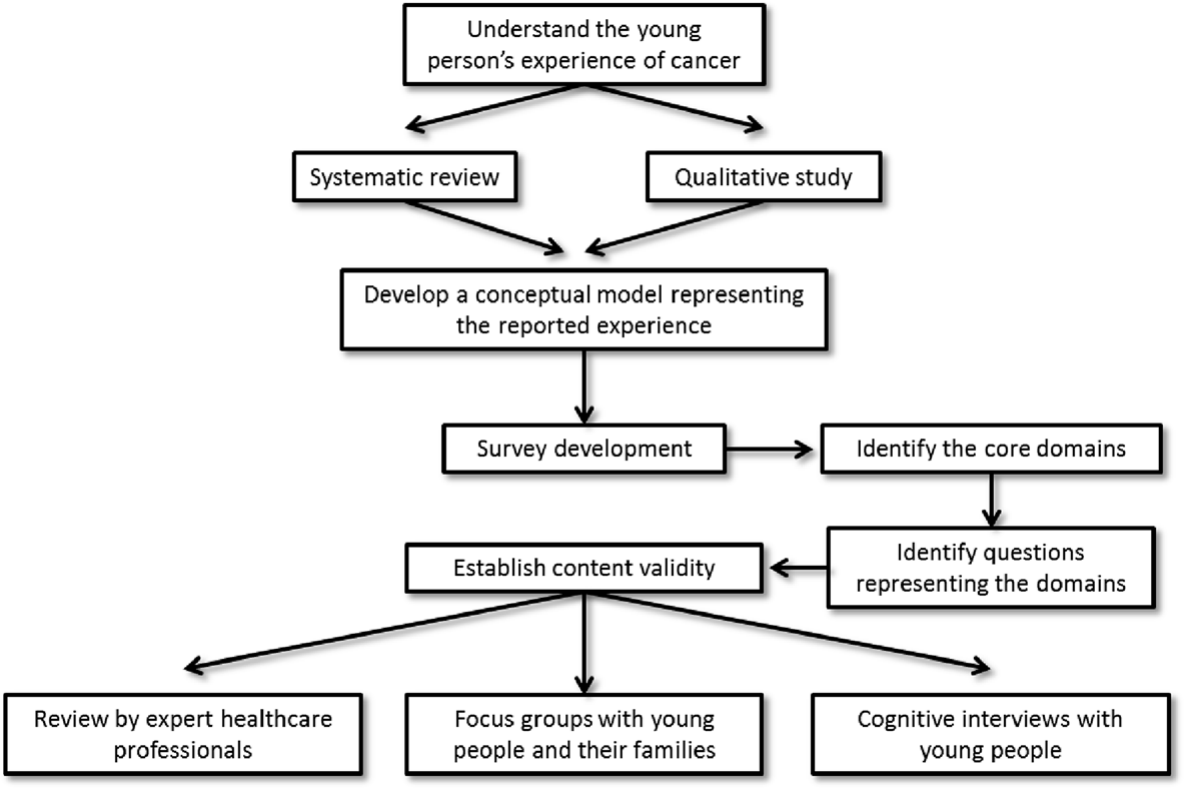
Schematic representation of the development of the BRIGHTLIGHT Survey

Described here is the development and validation of the content of the initial BRIGHTLIGHT Survey. In line with guidance for measuring patient experience [13, 14] the development of the survey occurred over a number of stages (Fig. 2):

1. Developing the content
2. Confirming the acceptability of methods of administration
3. Establishing comprehension

## Methods

### Stage 1: Developing the content of the BRIGHTLIGHT survey

The aim of the survey was to capture young people’s experiences of having cancer and to create questions to be able to quantify this. A systematic review of qualitative research on young people’s experiences with cancer identified 15 published studies [11]. A further four studies unpublished at the time of survey development in 2012 were also identified [10, 12, 15, 16]. The rich descriptions and quotes provided in these studies were used to generate questions and their response categories (see Table 1 for an example). A Google search identified other cohort and experience studies, restricted to the UK to ensure only culturally similar studies were identified. Relevant websites were accessed to obtain copies of questionnaires (Table 2).

**Table 1:** An example of a descriptive experience question developed for the BRIGHTLIGHT Survey

Why did you choose not to take part in the trial?	
1.I didn’t want to do it	
2.I was told about the trial at a difficult time	
3.Treatment in the trial was longer/didn’t want to have longer treatment	
4.Treatment in the trial was shorter/didn’t want to have shorter treatment	
5.Didn’t want to be part of an experiment	
6.I had too many things to think about already	
7.Was worried it would make me feel worse/my prognosis worse	
8.Would have had to go for more hospital visits	
9.Would have had to take more drugs/the trial would have increased the number of drugs I would receive	
10.Would have had fewer drugs/the trial would have decreased the number of drugs I would receive	
11.I didn’t understand what the trial was about	
12.I was not selected for the trial

**Table 2:** Sources of other Cohort and experience studies

National Survey Bank	
http://ukdataservice.ac.uk/get-data/key-data.aspx	
Centre for Longitudinal Studies	
http://www.cls.ioe.ac.uk/	
Avon Longitudinal Study of Parents and Children (ALSPAC)	
http://www.bristol.ac.uk/alspac/	
NHS Experience Surveys	
http://www.quality-health.co.uk/surveys	
http://www.nhssurveys.org/	
British Children’s Cancer Survivorship Study	
http://www.birmingham.ac.uk/research/activity/mds/projects/HaPS/PHEB/CCCSS/bccss/index.aspx	

Questions were developed from literature in all three searches by two members of the BRIGHTLIGHT Team (RMT/LAF). These were reviewed by the rest of the research team (a multidisciplinary expert panel, see co-authors) and subsequently distributed to experts additional to the research team for independent review to ensure the questions reflected the issues and the responses included the range of experience. Experts were selected based on their AYA and cancer expertise and/or knowledge of specific areas, i.e. patient choice and fertility. Differing opinions in survey content were resolved through discussion and consensus.

The item bank for the BRIGHTLIGHT Survey consisted of 15 domains with a total of 238 questions. The core domains identified for the initial survey included:

1. Experience before diagnosis
2. Diagnostic experience
3. Place of care
4. Contact with healthcare professionals
5. Treatment experience
6. Fertility
7. Involvement in clinical trials
8. Adherence
9. Communication and coordination of care
10. Education
11. Social support
12. Illness perception
13. Emotional state
14. Employment
15. Wellbeing and relationships

These were approved by an NHS Research Ethics Committee (study reference number: 11/LO/1718) as the template for further feasibility and validation testing.

Based on the information collected in the feasibility work [12] the aim was to develop a questionnaire that could be completed initially in a face-to-face interview in 40 min then subsequently online or through telephone interview in 30 min. Face-to-face interviews were chosen at wave 1 (6 months after diagnosis) as a way of engaging young people and to increase retention in future waves. The option of online or telephone interviews at waves two to five (12, 18, 24 and 36 months after diagnosis) were based on feedback from young people in the feasibility work, to give young people choice and thus increase retention in the study.

The item bank was calculated as being significantly longer than this and therefore the number of items was reduced to 169 across the 15 domains through discussion and consensus with key members of the research team. Young people were not involved in this stage because the focus was ensuring the questions related directly to the aims of the study (full protocol available from www.brightlightstudy.com). This version was used for feasibility testing (stage 2) and establishing validity (stage 3).

### Stage 2: Confirming the acceptability of methods of administration

To establish that methods of administration and the accompanying documents were acceptable to young people, focus groups with young people and their families were conducted to explore further the acceptability of administration methods. The objectives of the focus groups were to:

- Identify key challenges and concerns of taking part in the survey;
- Review drafts of supporting information, e.g. invitation letters and information leaflets;
- Identify concerns about the proposed survey process from consent through to interview completion;
- Confirm relevance of the proposed survey content;
- Explore young people’s perceptions of communication with the BRIGHTLIGHT Team through the 3 year study period.

### Participants

Four focus groups were conducted throughout England with a convenience sample of young people, parents and siblings (Table 3). Young people were eligible for inclusion if they had been diagnosed with a primary cancer between the ages of 13 and 24 years within the previous 3 years. Participants were recruited using a range of methods, including direct contact, charitable websites and social media. The focus groups were approved by an NHS Research Ethics Committee as part of the BRIGHTLIGHT patient and public involvement strategy. Verbal consent was taken from each participant at the beginning of each focus group and opportunities were given for participants to withdraw. Participants were assured of anonymity and confidentiality and afterwards received £30 token of thanks for their participation.

**Table 3:** Summary of participants in the focus groups

	Location	Participants	Gender	Age
1	Birmingham	Four young people	3 female, 1 male	14–22 years
2	Leeds	Two young people	1 female, 1 male	20–25 years
3	Cambridge	Five young people	2 female, 3 male	17–22 years
4	Cambridge	Three parents & 2 siblings	3 female, 2 male	No ages recorded. All siblings were younger

### Methods

Focus groups were held in non-healthcare settings around England and were facilitated by experienced moderators from Ipsos MORI and the BRIGHTLIGHT Team. The discussion was directed by a structured guide to ensure uniformity between focus groups but the moderators were reflexive to the flow of conversation in each group. Focus groups were digitally recorded and an assistant took field notes. Digital recordings were transcribed verbatim and analysed using qualitative content analysis.

### Findings

The findings were grouped into three topics: issues that had a direct effect on young people, issues relevant to the actual survey, and administration issues.

In general, young people and families expressed that BRIGHTLIGHT was extremely important and had they been eligible they would have been keen to participate. They were pleased to be given the opportunity to report their experiences of cancer and cancer services as they felt they did not often have this opportunity.

*“I think it’s useful to get people’s opinions about it [cancer services]… I just think it’s something that’s needed really. There’s really not much else”.* Female, Leeds

### Issues that had a direct effect on young people

Young people made general comments about the study as a whole but also comments related to three subthemes: motivation for taking part, potential barriers, and survey administration.

While young people were largely positive about participating in BRIGHTLIGHT in theory, they acknowledged that some participants may find the survey challenging. They proposed one of the main challenges to participation was cancer and treatment-related fatigue. An additional challenge was young people’s desire to have a ‘normal life’ away from cancer during periods when they were not experiencing severe symptoms or receiving treatment. Some participants suggested that this may adversely affect retention because some young people may not want to continue participation if they wanted to detach from their cancer experience.

*“People might just want to get on with their lives and not think about it” Female, Cambridge*

#### Young people’s motivation for taking part

The primary motivation for participation was a belief that BRIGHTLIGHT could improve services for other young people in the future. Many young people exhibited an altruistic opinion to help make receiving a cancer treatment a better experience for others in the future.

*“You’re getting involved, give something back so if the survey could probably help other people in the same situation in the future so it’s a good thing to be involved in”. Female, Birmingham*

Parents and siblings also felt that most young people would be happy to take part in the study, largely for the same reason of helping others in a similar situation in the future. Some believed that young people would benefit from being able to talk to a non-medical person about their experiences.

Some young people realised that the size and scope of the project meant: “*it could potentially change quite a few large-scale things”* (Male, Leeds). While this was clearly seen as the primary motivating factor, some young people felt that altruism alone would not be enough to encourage everyone to take part, especially at a time when they are facing the challenges associated with their cancer diagnosis.

*“When you’re really stressed with having found something out, like being diagnosed with cancer, to be honest, the last thing on your mind is doing random selfless things”. *Female, Leeds

There was also a concern that the research would not necessarily be used to improve services.

“*It could* be *put in to a file and no-one is going to do anything with it”* Female, Leeds

There was strong approval from young people about the amount of involvement young people had in the development of the survey. This was seen by some to be a *“huge selling point”,* as it would encourage young people to believe that the survey was more likely to be relevant to them.

*“I like the idea that it’s designed by young people; I worked for the university here for a while and we did a study there looking at academic feedback when we had it designed and run by young people and it was completely revolutionary to the university and I’m sure it’s just as revolutionary in the health care world”. Male, Birmingham*

#### Potential barriers

A key barrier to participation was identified as fatigue. Young people described limited levels of physical and mental energy as a key barrier that could make it difficult for young people to take part in the survey and to spend sufficient time thinking through their answers. For some, this might make it difficult to complete the survey initially and could also pose potential difficulties for completing the survey in one go.

*“You don’t always feel up to it”. Female, Cambridge*

Some young people mentioned they may struggle to engage with the survey during periods where they are feeling healthier as they would want to spend these periods having as normal a life as possible.

*“People might just want to get on with their lives and not think about it” Female, Cambridge*

In contrast however, others suggested that cancer would *“always be part of [my] life to an extent”* (Female, Birmingham), and consequently felt that the study will continue to feel relevant to them.

#### Survey administration

Young people thought that five interviews over three years were acceptable and would offer a good balance between sufficient numbers of interviews to allow interviewers to gather detailed information about those taking part whilst ensuring that the research was not too intrusive and burdensome to complete.

Young people were generally satisfied with the face-to-face interview process proposed; however they felt that the initial interview should not take place until five or six months after their diagnosis because it had taken at least two to three months to adjust to their diagnosis and to realise their cancer diagnosis.

*“The first few months you are trying to get your head around everything … right at the start it was just information overload”. Male, Cambridge*

Young people also suggested that parents can often take longer than their children to accept and adjust to their children’s diagnosis of cancer, making earlier contact for the study potentially difficult for them to cope with.

Young people expressed one of the advantages of the interview process was that the interview could take place at home as this would be a more comfortable setting than a hospital or medical centre.

*“The fact that the first [interview] is wherever you want it makes it a lot better … if you didn’t want it done in the hospital environment you could choose [not to]” Male, Cambridge*

Some of the older participants explained that they would not necessarily want their parents or other close friends or family to be present when they are being interviewed. A strategy to avoid this would be for the interview to be undertaken in a *“neutral place”,* something that has also been allowed for in the research design.

### Issues relevant to the actual survey

Young people and parents particularly valued questions about their general wellbeing and experiences rather than focussing just on their health and specific conditions. Young people understood the need for questions about their fertility and sexual activity and parents also thought it was fine to ask their children questions on these topics as it was relevant to their treatment and age. All the young people were happy that these questions could be answered anonymously so that the interviewer could not see the answers.

*“If you’re going to include it, I think that’s probably the most sensible way” [of asking those questions]. Male, Birmingham*

Young people expressed they would like some open questions included in the survey as these would allow them to contribute specific feedback on any issues that were important to them. It was also suggested that a variety of response formats should be used so there was some variety in the questionnaire. This was particularly important for the initial face-to-face interview.

*“I think if I had just had 40 min of interviews and it was like, answer A, B C or D… it would also feel like what’s the point of this person being here, because you could have just sent me the form and I could just fill it in, to be ticking some boxes for you”. Male, Leeds*

Some parents also commented on the need for the questions to be phrased in a sensitive manner, especially in circumstances where prognosis was poor. They felt it was important that in these cases respondents were not asked to think about the future. As a result routing for some of the questions that ask young people to think about their life going forward was amended so that these questions were not asked of those in contact with the palliative care team.

### Administration issues

There were four administration-related issues that were discussed: the invitation letter and information leaflet; the consent form; the study website; and survey updates.

#### The invitation letter and information leaflet

Draft versions of the advance letter and leaflet text were well received by young people. In general, they believed the information provided was *“clear”* and *“thorough”,* without being too much; they were clear that being presented with anything too text heavy would put them off reading it. There was also widespread support for the logo used for the study, which had been developed by young people [17]. The logo enabled the study to be easily identifiable. Suggestions for improvements were incorporated into the documents for the final focus group. This included adding more colour and changes to formatting. Participants in the final groups were satisfied with the revised letter and leaflet.

*“I think it all makes sense. The sentences are quite clear”. Male, Cambridge*

*“The letter… I think it is quite easy to understand”. Female, Birmingham*

Many young people said that they might not have the energy to read the leaflet immediately. Nevertheless they believed the leaflet would be useful to refer to if they had any specific questions about the study. Young people commented that big blocks of text may discourage them from reading. They suggested breaking up the text with photographs and pictures relevant to the study.

#### The consent form

Young people said that the consent form was clear and well designed. However, they wanted to be able to tick boxes rather than write their initials as this would be quicker and easier for them to manage (initialling is recommended in the UK by the National Research Ethics Service: http://www.hra-decisiontools.org.uk/consent/). They also expressed the need for clarity of which points were compulsory for participation and which were optional (e.g. informing their General Practitioner about participation).

*“It’s much better than other forms you have to fill out. It is quite well laid out”. Male, Cambridge*

#### Study website

Young people were largely enthusiastic about online options for taking part in the study and receiving reminders and information about the study in the future. Many young people noted the convenience of online options because they regularly used social networking sites such as Facebook and Twitter, and many had access to smart phones allowing them to access the sites while on the move.

*“I think a lot of people use Facebook for updates. You could put updates up that way”. Female, Cambridge*

In addition, parents were also keen on some online options such as a chat room where they could discuss their experience with others in a similar situation. While paper materials could easily be misplaced, it was felt that online materials were easily accessed from a number of locations. Young people also believed that online materials were easier to browse through than paper versions as they could be less linear and allow users to dip in and out of materials rather than having to tackle them all at one sitting.

#### Survey updates

Young people viewed survey updates positively and suggested that they could help keep respondents engaged with the survey and would be keen to receive them. They suggested that the updates might include details of the emerging findings or updates about how the research was being used.

*“Yeah which I assume will be published in medical journals etc., which unless you’re affiliated with the university you can’t really get to without paying; if you could send one to each TCT [Teenage Cancer Trust] ward maybe as you’re going along it could probably keep them interested, certainly it would keep me interested in it…..just so you could see that something was actually happening as a result of the study” Male, Birmingham*

*“Maybe each month they could publish the result of a different question in the survey. So you could find out what people think”. Female, Cambridge*

Some respondents felt updates on how the research was being used was particularly interesting and would provide further reassurance that the research results were being implemented and taken seriously. Another suggestion was that updates could include feedback from people taking part in the survey about the survey process because this could help to influence those who were unsure about taking part.

*“If you’ve got [someone] within the cohort saying “it’s not as easy as it sounds” I think that potentially could help”. Male, Birmingham*

### Stage 3: Establishing comprehension of the BRIGHTLIGHT survey

The content of the survey had been developed by working with young people and rigorously reviewed by healthcare professionals and academics with expertise in cancer and patient experience. However, it was important to ensure the questions were not only a good reflection of young people’s experience but the questions could be understood by young people. Cognitive interviewing is one approach to establish whether questions are understood, whether participants have the knowledge to complete questions and consistency of interpretation [18]. Specifically, several key areas were tested (Table 4).

**Table 4:** Key areas test in the cognitive interviews

Key area	Examples
*Comprehension*	Request clarification, e.g. on the meaning of words, phrases, an entire question, or whether certain things should be in- or excluded?
	Recall/judgement, are they able to think back? Do they find this difficult/easy?
	Did respondent have trouble remembering the information?
	Watch out if respondent is not answering with the information the question is asking about, i.e. misconceiving the question.
*Response*	Social desirability, i.e. responding according to what they think people will want to hear/expect rather than their true opinion.
*Other factors*	Do the questions cover all circumstances or are any responses missing?
	Is there any indication that the question may be too long or wordy
	Does the routing work to guide respondents through the questionnaire; are they asked any inappropriate questions?
	Any issues/problems with questions being too sensitive or any concerns?
	Any age/cancer type/treatment issues with answering the questions?

A number of previously validated questionnaires were to be administered within our survey package. These included the PedsQL™ generic module [19], the Hospital Anxiety and Depression Scale [20], the Brief Illness Perception Questionnaire [21] and the Multidimensional Scale of Perceived Social Support [22]. No publications reporting cognitive testing of these questionnaires with young people were identified and therefore these were also included in this stage.

### Participants and settings

Participants recruited for cognitive testing were a convenience group contacted via social media sites such as Teenage Cancer Trust Facebook page, JimmyTeens Facebook page and Twitter. Young people were eligible for inclusion if they had been diagnosed with a primary cancer between the ages of 13 and 24 years within the previous 3 years. A purposive sample was selected from those responding based on gender and age to ensure representation (LAF/AS). In addition, we selected specific groups with potential cognitive difficulties as a result of treatment, e.g. central nervous system tumours, or potentially had a different perception of cancer, e.g. young people with a low risk malignant melanoma. This was to ensure that the survey was appropriate for those who may have experienced cancer-related cognitive affects and those whose experience of cancer care was relatively little. Participation in the interview was taken as consent; however, for those under the age of 16, written consent was gained by a member of the BRIGHTLIGHT team from a parent or guardian for them to take part.

A total of 41 young people responded to the adverts from which a sample of 23 young people were selected for cognitive interviewing, these consisted of 14 female and 9 male aged 14–24 years (Table 5; we were unable to recruit any young people aged 13).

**Table 5:** Participants of the cognitive interviews

	Total (*n* = 23)
	n (%)
Gender	
Male	9 (39)
Female	14 (61)
Current age (years)	
14–15	2 (9)
16–19	10 (43)
20–24	11 (48)
Age at diagnosis (years)	
14–15	10 (43)
16–19	7 (30)
20–24	6 (26)
Tumour site	
Brain tumour	2 (9)
Lymphoma	6 (26)
Leukaemia	10 (43)
Other	5 (22)

### Methods

Interviews were conducted with young people in two phases. A total of 23 cognitive interviews were completed, with eight in the first phase and 15 in the second. Changes to the survey were made between the two phases of the cognitive interviews to test the amendments made following the first interviews. All questions were tested more than once although new questions were prioritised over validated questions, and problematic questions were tested among a larger group. Interviews were conducted by telephone by experienced researchers from Ipsos MORI and lasted between 40 and 60 min. Young people were aware that they could stop the interview at any time, although none did. Participants were given £30 voucher afterwards as a token of thanks for participating.

It is relevant to note that those taking part in the cognitive interviews came to the survey with much less information than real participants in the study would have. Participants in the main study would be given more detailed information about BRIGHTLIGHT in advance, and would also have the opportunity to speak to healthcare professionals involved in recruitment so the context and circumstances would be different.

### Findings

The questionnaire was well received by respondents and additionally there were no problems in cognitive understanding of the 14 year olds negating the problem of not recruiting any young people aged 13 years. In general, they reported the topic areas covered as important and relevant. Feedback related to specific changes to individual questions. Details of the identified problems and subsequent solutions are shown in Table 6.

**Table 6:** Results of the cognitive interviews and changes made to the survey

Section of the survey	Problem identified	Change made to the survey
Demographic questions	No response available for young people deferring a year of education to have treatment	Added response: *Taking a break from education*
PedsQL™	Instructions ask respondents to reflect on the past month. Some respondents found their situation varied too much in a month (i.e. chemotherapy cycles) so they wanted to put two different answers to cover how they felt over the past month	Used the acute version rather than standard version, which reflects on the past 7 days.
	The *‘How I get along with others*’ section:	No changes could be made to this section
	● Older young people who were working did not necessarily compare themselves just to their age group/other young people, but their colleagues and other young adults generally;	
	● Young people with children said they often did not see many other young people their age and if they did then they also did not compare themselves with young people but other parents. A suggestion was made to ask how they got on with family rather than peers.	
	The ‘*About work/studies*’ section was problematic for those who were not in work, or education in the past month.	Additional text added to reflect other life stage options (school, training, university). As advised by the author, if this is not completed, the total score will be calculated without the domain included.
Before Diagnosis	Difficulty in answering a question related to the time between symptom and diagnosis if some symptoms were earlier than those that they thought were related to cancer.	Amend the wording to ask respondents specifically when they noticed a symptom they thought might be cancer.
		Amending a question to make it clear that the question was asking for experience about when they first thought something was wrong.
		Amending the routing for a question so that those who went to A&E were not asked whether this contact was NHS or private.
		The word ‘consultant’ was added to the code ‘hospital doctor’ as this is how they were referred to.
		The code ‘*Hodgkin's disease*’ was amended to ‘*Hodgkin's disease/lymphoma*’ while the abbreviation ‘*A.L.L.*’ was also added to the code ‘*Acute lymphoblastic leukaemia*’ to make sure the options covers all the possible phrases that respondents were using.
Place of care		Simplified the wording of questions related to choice.
		Routing the text so that the phrase ‘*Other than visits to A&E (Casualty)*’ is only shown if they have been to A&E recently.
Treatment	Unable to answer a question related to treatment choice when there was no choice.	Adding a code ‘*Only one possible treatment was available*’.
	Did not understand the term ‘complementary therapy’.	Added a definition
Clinical trials	Did not understand what a clinical trial was.	Added a definition
Communication and coordination of care	Did not understand the term ‘nurse specialist’.	Added a definition
Education	Most respondents chose the final response for a question asking about the amount of time off school etc. because of being unwell.	Changes were made to the time period responses.
Employment		Changed the wording from ‘why’ to ‘for what reasons’ [have you not told your employer…]
		The words ‘my’ and ‘treatment’ were added to questions on the effects of cancer on employment.
		Changed the wording of ‘why’ to ‘for what reasons’ [have you decided not to go for this job].
		A code ‘want to be/stay close to my friends and/or family’ was added to the question about changing job.

*PedsQL* Pediatric Quality of Life Questionnaire, *A&E* accident and emergency

Prior to testing there was concern from healthcare professionals advising on the survey that respondents may have had reservations about answering questions related to fertility due to their sensitive nature; however, no respondents raised any concerns with these questions. Respondents understood why these questions were included and said it was important that they were asked.

A number of problems were identified with the validated questionnaires incorporated into the survey, but limited changes could be made without psychometrically testing the revised instrument. While the PedsQL™ generic module was selected as the best available measure of quality of life [12], a limitation noted was the lack of variation in items to reflect developmental changes from child to adulthood. This was especially so in the role (school) domain, which refers to ‘school’ in the child and teen versions and work or college in the young adult version. For those not in education or employment this aspect of the instrument is less relevant. When the Survey is administered through computer there is the ability to disable these questions if young people were not in education or employment, but this could affect questionnaire acceptability in paper versions.

Young people were confident in answering questions in the Brief Illness Perception Questionnaire and the Multidimensional Scale of Perceived Social Support and did not report any issues of sensitivity in answering the questions. However, the Ipsos MORI researchers found them to be the most challenging to ask. The rest of the questionnaire covered factual topics that all the respondents were willing to answer, probably because they had become used to talking about their health and treatment. These two sections in contrast, asked them to think about how they were supported and how positive they were about their treatment and outlook.

### Discussion

Described here is the development of a patient experience survey designed specifically for adolescents and young adults with cancer. Unlike reports on the development of other questionnaires, we explored and tested not just the content of the survey but also issues related to its administration. We found that while some service and cancer-related issues were important to young people, they clearly wanted to focus on the young person-related issues, such as careers and relationships [10]. Importantly for a longitudinal study, this was particularly apparent after completing treatment.

While the patient experience surveys in the UK health service are valuable they utilise standard content and method of administration, which reflects the specific purpose of supporting current healthcare policy, mainly addressing the needs of older adults or parents of children. We sought to go beyond this and focus on the experience of the young person. To this end, the role of patient and public involvement (PPI) has been critical not just in the development of the survey but in the design of the study as a whole. Young people and healthcare professionals were involved from the onset in feasibility studies [10, 11, 23, 24] and young people have been instrumental to survey development and working in an advisory capacity as the study has progressed [17]. Initial data from BRIGHTLIGHT suggests a higher than anticipated uptake among those offered participation and higher than anticipated retention; we suspect this is because study design and survey content has been rigorously tested and facilitated by an extensive PPI strategy.

This study has a number of limitations. The BRIGHTLIGHT Survey does not include end-of-life questions. This was a purposeful decision as little is known about end of life experiences in this population and it was felt that a survey may not be the best study design in this instance. An in-depth exploration of end of life experiences is currently underway [25], which may inform future survey content. However, the experience of those approaching end-of-life will be captured through the longitudinal nature of the study as they continue to participate. A further limitation is the respondents who participated in the focus groups and cognitive interviews opted-in to the research. As such, they are perhaps a group that may be atypical from the general AYA cancer population, e.g. those who are most comfortable speaking about their diagnosis, treatment and care, higher level of education. However, we included young people with a range of diagnoses, in different parts of the treatment trajectory, with a variety of hospital experience who were from a range of geographical locations and therefore they could be considered a good proxy for the views of the young people who are eligible to participate in BRIGHTLIGHT. Furthermore we specifically included young people with potential cognitive impairment, such as those with whole brain radiation and central nervous malignancies. This suggests the survey would be accessible to young people with lower education attainment or treatment-related cognitive abilities. Finally, the focus groups had a small number of participants potentially not capturing the opinion of the wider cancer community. This did enable us to explore specific issues in more depth, which may not have been possible in a larger group.

While most reports of questionnaire development focus on content and validation, it needs to be remembered that the questionnaire or survey is only one part of the study design. If accompanying letters, information and methods of administration are not acceptable then this will impact on the success of data collection and in the case of longitudinal research, retention. BRIGHTLIGHT currently has four waves of data collection underway (5, 12, 18 and 24 months after diagnosis). Early indicators suggest greater than 70 % retention. We believe that this is in part due to the engagement of young people in study development, ensuring the content of the Survey reflects their experiences, is understandable and that the survey methods are age-appropriate to this group.

### Conclusion

We have developed a measure of patient experience for young people with cancer; the survey was developed with young people for young people. Key design features of the BRIGHTLIGHT Survey include: i) content reflecting young people’s experience; ii) face-to-face administration in the location of choice at wave 1; iii) online or telephone administration at subsequent waves; iv) complex routing so young people are only asked questions relevant to their current life situation; v) ‘Pull-through’ function so young people are able to reflect in the current wave on responses given in previous waves. The data gathered through the survey will begin to tell us what it is truly like to be treated for cancer as young person and whether specialist cancer services for young people are appropriately designed so as to offer the maximum chance of physical and psychosocial recovery.

The BRIGHTLIGHT Surveys will be available to download from http://www.e-lucid.com/ Wave 1, as described in this paper, contains the core domains that are included in subsequent waves; however additional questions have been included in wave two onwards to measure the impact of fatigue, acute toxicity and explore young people’s concerns about recurrence. These were identified through collaboration with the Young Advisory Panel, the user group working on BRIGHTLIGHT and developed in the same manner as wave 1 questions.

## Abbreviations

NHS: National Health Service
NIHR: National Institute for Health Research
PPI: Patient and public involvement
TYA: Teenager and young adult
UK: United Kingdom
